# Supplementary material for: Incidence of the Brownian Relaxation Process on the Magnetic Properties of Ferrofluids
Source: Nanomaterials (Basel). 2024 Apr 5;14(7):634. doi: 10.3390/nano14070634 (PMC11013599; doi:10.3390/nano14070634)
Supplement: Supplementary file 1 [file nanomaterials-14-00634-s001.zip › nanomaterials-2907119-SI.pdf]

Supplementary Materials: Incidence of the Brownian relaxation process on the magnetic properties of ferrofluids

Lili Vajtai,† Norbert Marcel Nemes,*,‡,¶ Maria del Puerto Morales,§ Kolos Molnár,‖,⊥,# Balázs Gábor Pinke,‖ and Ferenc Simon†

†*Department of Physics, Institute of Physics, and HUN-REN-BME Condensed Matter Research Group, Budapest University of Technology and Economics, Műegyetem rkp. 3., H-1111 Budapest, Hungary*

‡*Departamento de Física de Materiales, Universidad Complutense de Madrid, 28040 Madrid, Spain*

¶*Institute for Solid State Physics and Optics, HUN-REN Wigner Research Centre for Physics, PO. Box 49, H-1525 Budapest, Hungary*

§*Department of Nanoscience and Nanotechnology, Instituto de Ciencia de Materiales de Madrid (ICMM-CSIC), 28049 Madrid, Spain*

‖*Department of Polymer Engineering, Faculty of Mechanical Engineering, Budapest University of Technology and Economics, Műegyetem rkp. 3., H-1111 Budapest, Hungary*

⊥*HUN–REN–BME Research Group for Composite Science and Technology, Műegyetem rkp. 3., H-1111, Budapest, Hungary*

#*MTA-BME Lendület Sustainable Polymers Research Group, Műegyetem rkp. 3., H-1111 Budapest, Hungary*

E-mail: nmnemes@ucm.es

Abstract

This Supplementary Material is organized as follows. First, we give a detailed description of the sample preparation method, then we summarize the results of the sample size characterization. This is followed by the summary of the calculation of converting magnetization units between SI and CGS as well as between the values normalized to the mass of iron and magnetite. In the next section, there is a detailed calculation of approximate sample sizes based on room temperature magnetic hysteresis data, whose results can be found in the main text. Next, the concentration dependence of the observed effects is investigated with an emphasis on the comparison of zero-field-cooled and field-cooled magnetization. The particle size dependence is also looked at in a similar way. Finally, the results of Differential Scanning Calorimetry and the determination of the freezing and melting temperature ranges are also detailed.

Sample preparation and characterization

The nanoparticles were obtained following the coprecipitation protocol described by Massart,[?] by introducing small modifications to control particle size between 6 and 14 nm. The synthesis of Fe_3O_4 magnetite nanoparticles was carried out by mixing 488 mL of a ferrous-ferric solution, $FeCl_3 \cdot 6H_2O$ (0.09 mol) (27% purity, $d = 1.26$ kg/L, MW = 162.21 g/mol, Sigma-Aldrich) and $FeCl_2 \cdot 4H_2O$ (0.045 mol) ($\geq$99% purity, MW = 198.81 g/mol, Sigma-Aldrich), with 75 mL of NH_4OH (28.0-30.0% purity, $d = 0.9$ g/mL, MW = 35.05 g/mol, Sigma-Aldrich). The precursors were added and magnetically stirred. Subsequently, it was allowed to cool to room temperature and washed three times by magnetic decantation with distilled water. To obtain larger nanoparticles, the sample was subjected to a heat treatment at 90 °C for 1 hour before washing.

The precipitate was subjected to an acid treatment to improve the colloidal stability of the nanoparticles. To do this, 300 mL of 2M HNO_3 nitric acid (65% purity, $d =$ 1.68 g/cm, MW = 404.00 g/mol, Sigma-Aldrich) were added to the nanoparticles and kept stirring for 15 min. After that time, the supernatant was discarded by magnetic decantation and 75 mL of 1M $Fe(NO_3)_3$ iron nitrate ($\geq$99% purity, $d = 1.37$-1.41 g/mL, MW = 63) and 130 mL of distilled water were added. The sample was boiled for 30 min while stirring, then it was allowed to cool to room temperature and treated again with 300 mL of 2 M HNO_3 for 15 min. The resulting nanoparticles were washed 3 times by magnetic decantation with acetone. Subsequently, the nanoparticles were resuspended in 90 mL of distilled water. The acetone was completely removed with the help of the Rotavapor at 60 °C and 556 mbar (Rotation evaporator Laborota 4011, Heidolph, Schwabach, GER).

Particle sizes were measured with a JEOL JEM 1011 transmission electron microscope (Peabody, MA, USA) with Gatan ES1000Ww camera (Pleasanton, CA, USA), and the recorded images are depicted in Figure 1 (6-nm sample) and 2 (13.5-nm sample). Based on these images, particle size histograms were created (Figure 3), and Gaussian fits were performed. As a result, particle size data were obtained as 5.8$\pm$1.5 nm for the

6-nm sample, and 13.2±4 nm for the 13.5-nm sample.

Hydrodynamic sizes in acid (pH = 3) were measured with dynamic light scattering, resulting in 40 nm (PDI: 0.13) for the 6-nm sample and 76 nm (PDI: 0.17) for the 13.5-nm sample. As expected, the hydrodynamic sizes are much larger than the nominal ones, affecting the dynamics of Brownian relaxation.

Magnetization normalization and conversion

The mass magnetization in CGS units (emu/g) can be conveniently converted into the SI (volume) magnetization units of A/m using that 1 emu $= 10^{-3}$ Am^2 (NIST link: https://www.nist.gov/document/magneticunitspdf). Correspondingly, for the volume magnetization, we obtain 1 emu/cm^3 $= 10^3$ A/m. As a result, for magnetite with 80 emu/g Fe_3O_4 and density 5.170 g/cm^3, we obtain 413 kA/m.[?]

When normalized by the Fe mass, the emu/g Fe values have to be multiplied by 0.723 to obtain emu/g Fe_3O_4 values.

Magnetic particle size evaluation based on the Langevin function slope

The main text showed fits to the room temperature magnetization data using the Langevin function. This function has two parameters: the saturated magnetization, M_s, and the particle magnetic moment, μ_{part}. The latter is essentially a horizontal scaling factor, which is in turn sensitive to the particle size. The nature of the fits is such that it is more sensitive to the value of M_s than to μ_{part}. However, μ_{part} can be also determined from a numerical derivative of the data as shown in Figure 4.

The figure shows a comparison between the derivative of the original data and that of the fitted Langevin functions. Clearly, the derivative of the Langevin functions is below for both types of samples. In order to match the two types of data, μ_{part} has to be increased by about a factor 2 for the 6 nm sample and by a factor of about 6 for the 13.5 nm sample. As the main text discusses, these increased μ_{part} values give rise to a more realistic magnetic particle size.

Concentration dependence of the observed effects

The main text discusses results on samples with 3 mg/mL concentrations. In the following, we discuss the effect of the varying concentrations (Figure 5). The major result can be summarized as follows: the observed behaviors do not show any concentration dependence for 10 and 20 mg/mL but changes start to appear for 30 mg/mL.

The error of the Fe amount estimate is significant (which can be traced back to the large error of the mass measurement): the smallest value of M_{sat} is 49.32 emu/g Fe for the 10 mg/mL sample, while it is 110 emu/g Fe for the 20 mg/mL sample with no apparent systematic dependence of these values on the concentration.

In Figure 6 we show the field-cooled (FC) and zero-field cooled (ZFC) magnetization curves for the 13.5 nm sample for 4 different concentrations: 3, 10, 20, and 30 mg/mL. The data were normalized by the value of the saturated magnetization.

In Figure 7 we show the thermal hysteresis curves for 4 concentrations: 3, 10, 20, and 30 mg/mL. Data were normalized by the value of the saturated magnetization.

Systematic particle size-dependence

Figure 8 shows the FC vs. ZFC measurements for the different particle sizes. The observations that are reported in the main text are reproduced with a systematic particle size dependence: the FC data drops at the water freezing point for the small diameter samples and it is continuous for the ZFC measurements. However, it increases for both the FC and ZFC measurements for the larger-diameter sample, which is a benchmark of the Brownian relaxation. The small-diameter samples are characterized by a blocking behavior at low temperatures (blocking temperatures are indicated by arrows), whereas the temperature-dependent magnetization is ”flat” for the larger-diameter samples.

Figure 9 shows the thermal hysteresis effect for the varying sample diameters of 6, 8, 10.6, and 13.5-nm samples at the 10 mg/mL concentration. The diameter dependence again shows several systematic diameter-dependent effects: the small diameter samples are characterized by a blocking behavior at low temperatures, whereas the temperature-dependent magnetization is ”flat” for the larger diameter samples. At the water freezing point, the cooling magnetization data is above the warming for the small-diameter samples whereas this behavior is reversed for the larger-diameter ones. Both observations occur for the intermediate sample diameters, too but with reduced effect compared to the two extremal behavior at the 6 nm and 13.5 nm samples.

Results of Differential Scanning Calorimetry

When performing Differential Scanning Calorimetry measurements, the heat flow is recorded, which is needed for keeping a slow, uniform cooling of the system. Data were taken with a ramp rate of 5 K/min, a slower rate did not influence the measurement. The heat flow value was normalized by the sample mass depicted as a function of temperature (Figure 10).

Transition temperature ranges depicted in the main text were determined the following way: in the DSC data shown in Figure 10 the melting and freezing transitions appear as peaks. The starts and ends of these peaks can be approximated as linear, and the transition points are determined as where these lines intersect the baseline. These melting and freezing temperature ranges are shown in the main text and more details are provided in the Supplementary Materials.

To examine the effect of evaporation on the heat flow data, the measurement cycle was repeated for the 6-nm sample (Figure 10a). There is no visible change in the shape of the measured curves, except in the freezing peak, but that is clearly undersampled, so the deviation probably stems only from this. Its start and end, which are relevant for determining the transition points, however, do not suffer from this problem. Therefore we concluded, that there is no considerable evaporation during the time scale of the measurement, which would modify the results. As this process is rather tied to the carrier fluid than to the nanoparticles, we generalized this conclusion for the other samples as well.

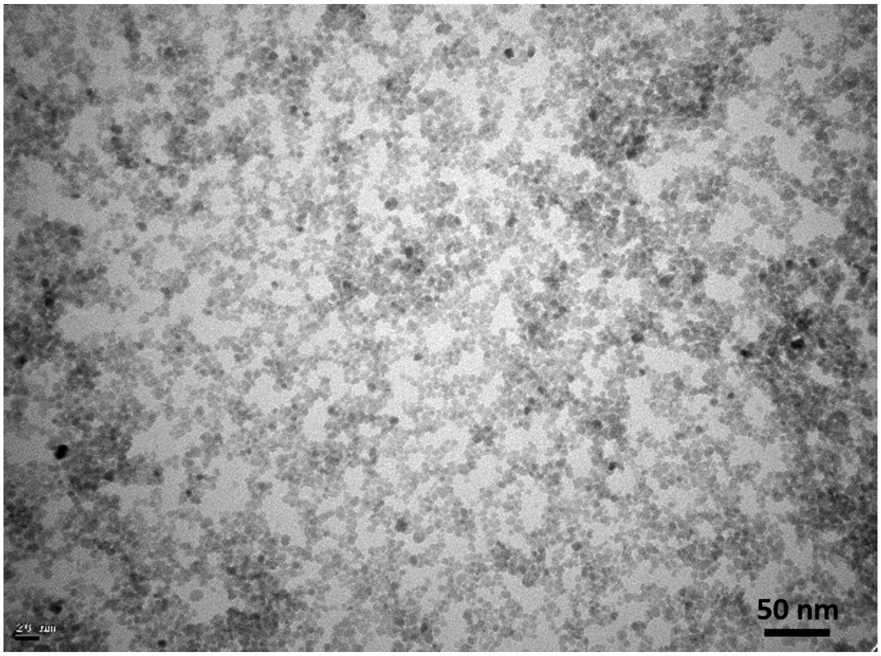

Figure 1: TEM image of the 6-nm sample.

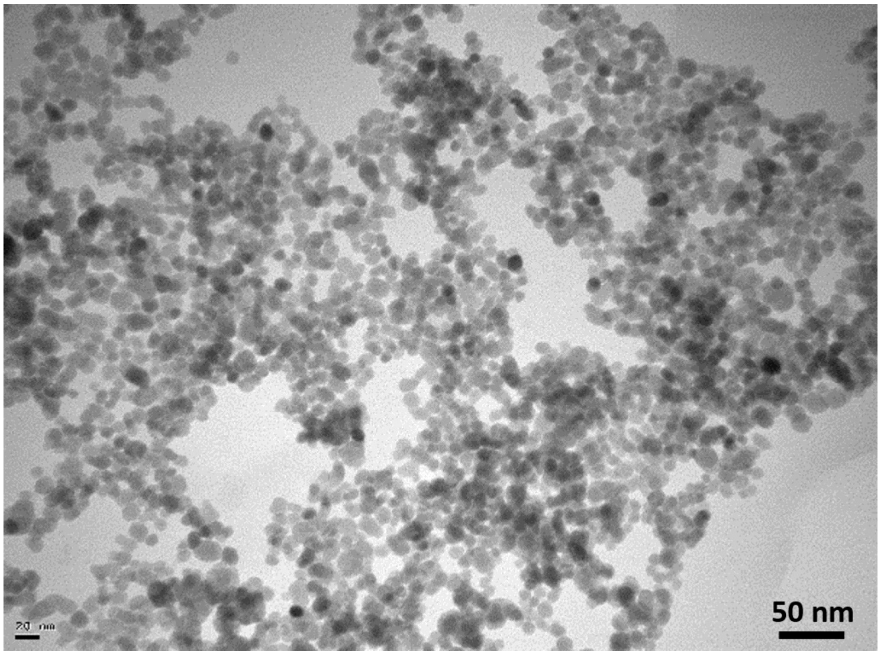

Figure 2: TEM image of the 13.5-nm sample.

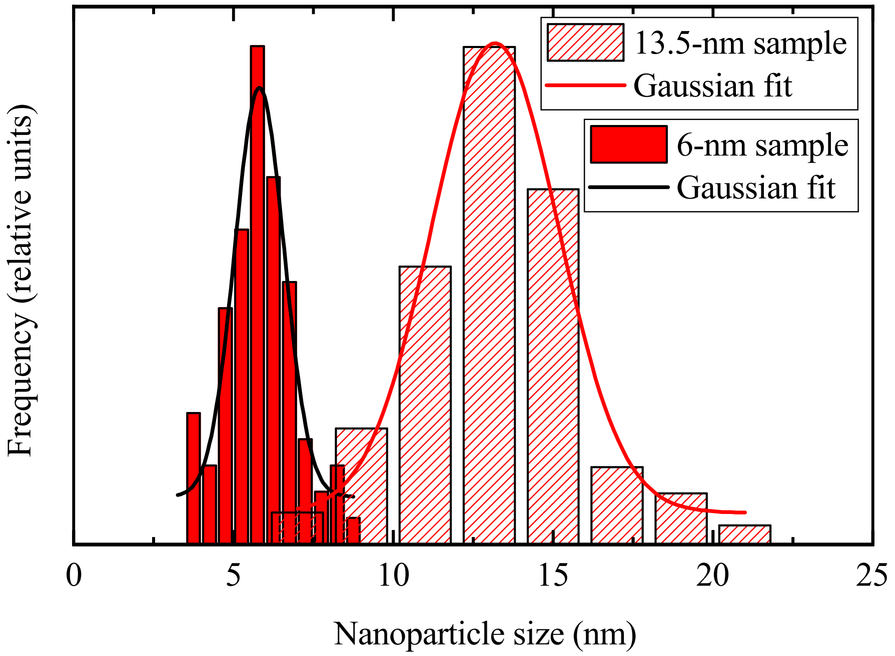

Figure 3: Histogram of particle sizes based on the TEM images of the 6-nm and 13.5-nm samples along with Gaussian fits.

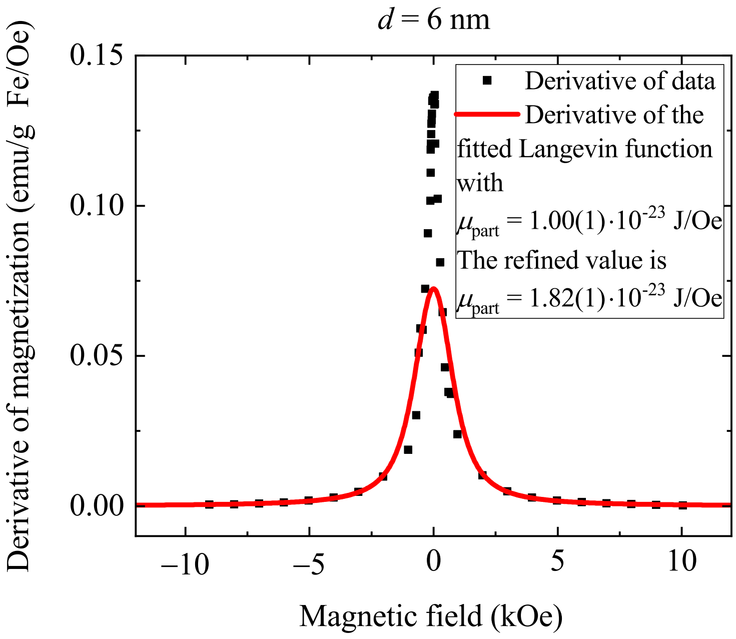

(a)

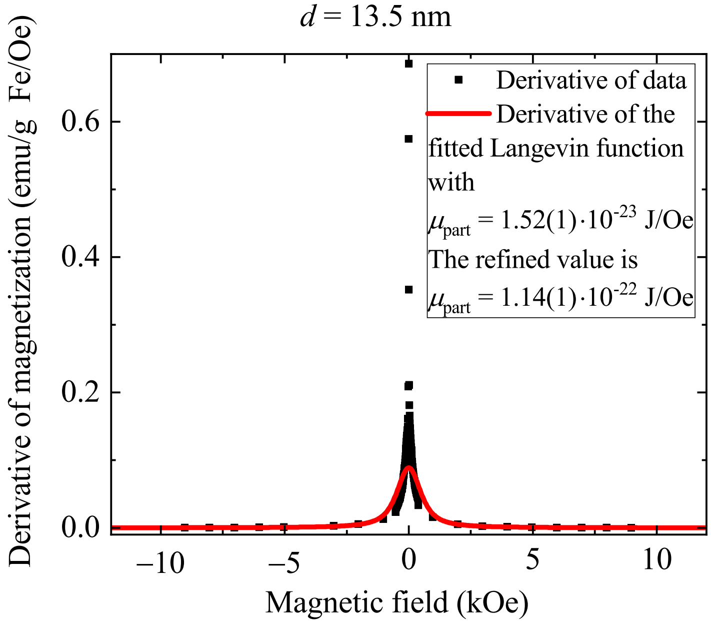

(b)

Figure 4: Derivative of the magnetization curves for the two types of magnetite samples recorded at 300 K: containing 6-nm particles (a) and 13.5-nm particles (b). Solid red curves show the derivative of the fitted Langevin curves. Clearly, the derivative of the fits is below that of the data. This indicates that the μ_{part} values obtained from the Langevin fits are below that obtained from matching the $B = 0$ slope. The latter gives rise to significantly increased values of μ_{part} as discussed in the main text.

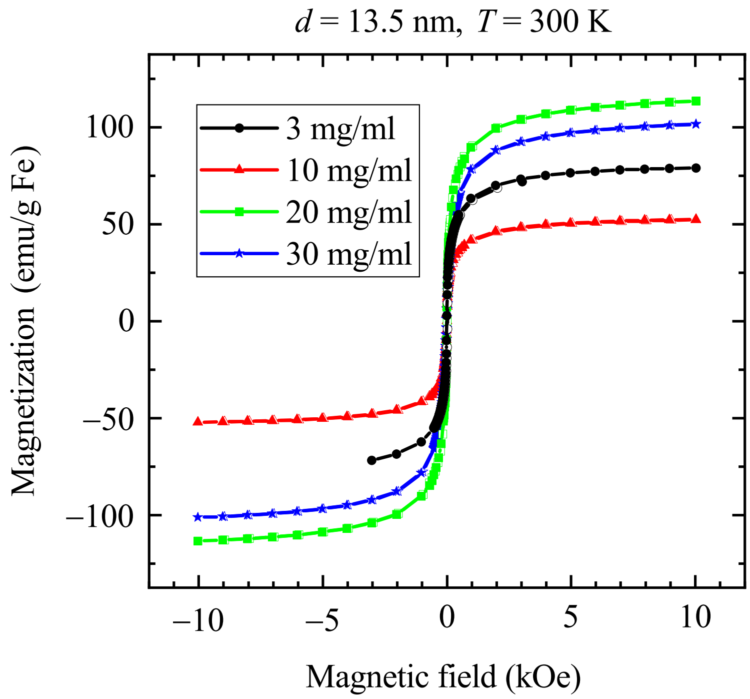

Figure 5: Magnetic field-dependent magnetization curves for the 13.5 nm sample for 4 different concentrations. The different saturated values are probably due to the errors in the estimate of the Fe concentration.

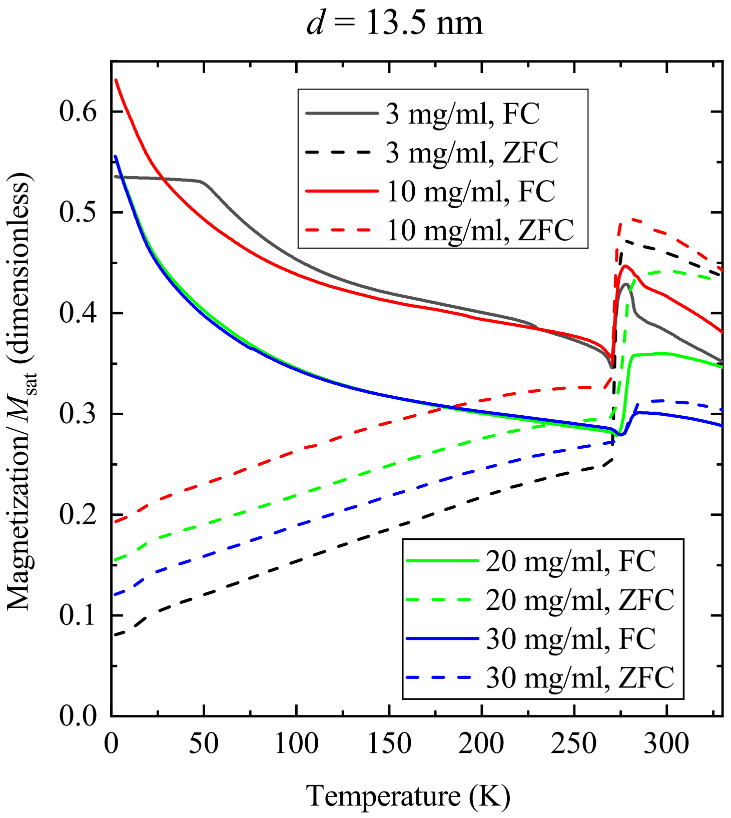

Figure 6: Comparison of field-cooled (FC) and zero-field cooled (ZFC) magnetization curves for the 13.5 nm sample for 4 different concentrations after normalization with the saturated magnetization.

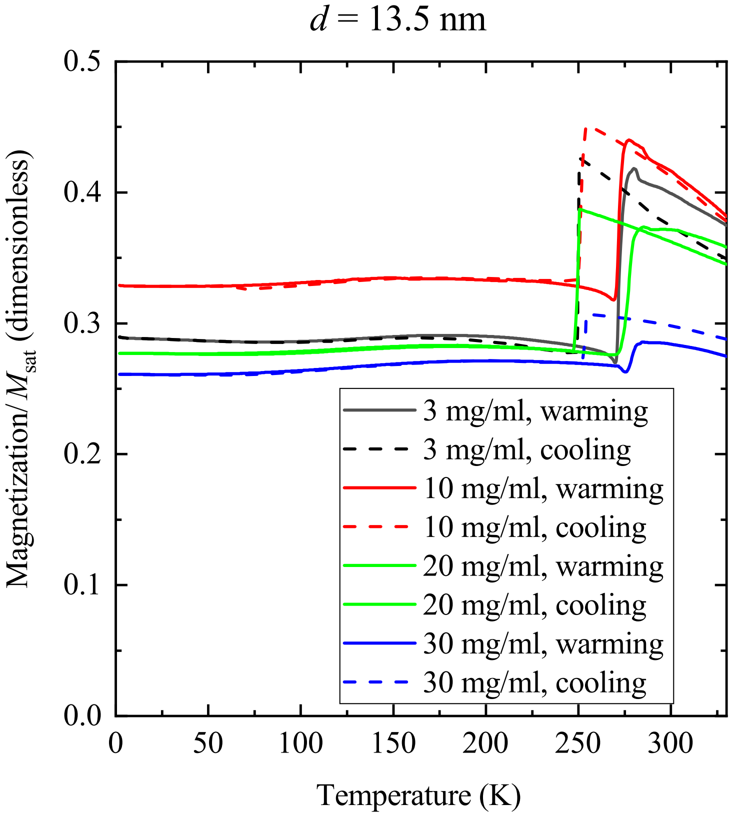

Figure 7: Temperature-dependent thermal hysteresis curves for the 13.5 nm sample for varying concentrations. The measurement was performed at 100 Oe while cooling and warming the sample. The original Fe mass-normalized magnetization data were further normalized by the value of the saturated magnetization due to an error in the Fe amount estimate. This puts all curves onto similar values. Note that for the highest concentration, the thermal hysteresis curves markedly differ from the lower 3, 10, and 20 mg/mL values.

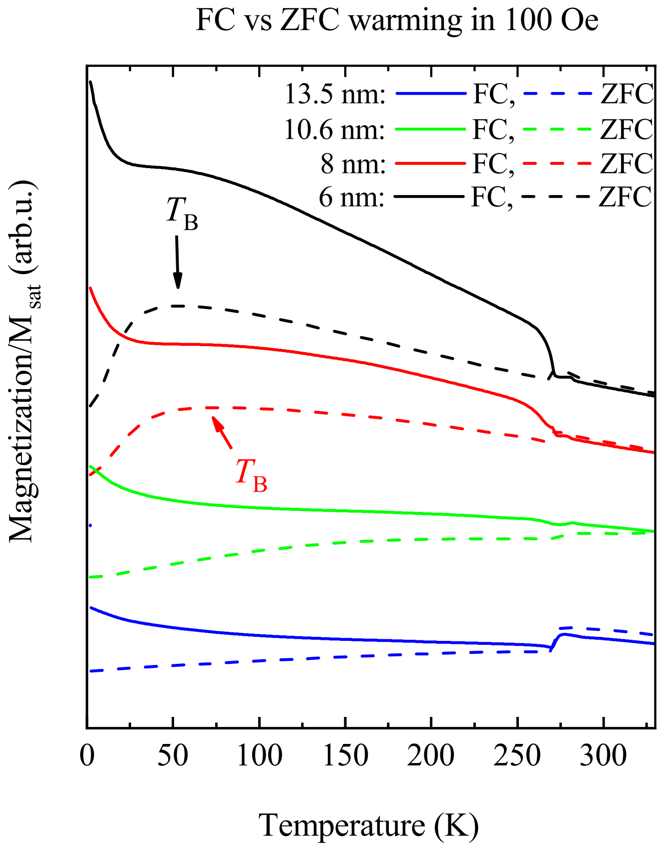

Figure 8: Particle size dependence of the FC vs ZFC experiments (measured at 100 Oe) for the 6, 8, 10.6, and 13.5-nm diameter samples for the 10 mg/mL concentration. The measured magnetization values are normalized by the saturated magnetization value at 330 K and are offset for clarity. Arrows indicate the blocking temperature for the samples which show the Néel behavior.

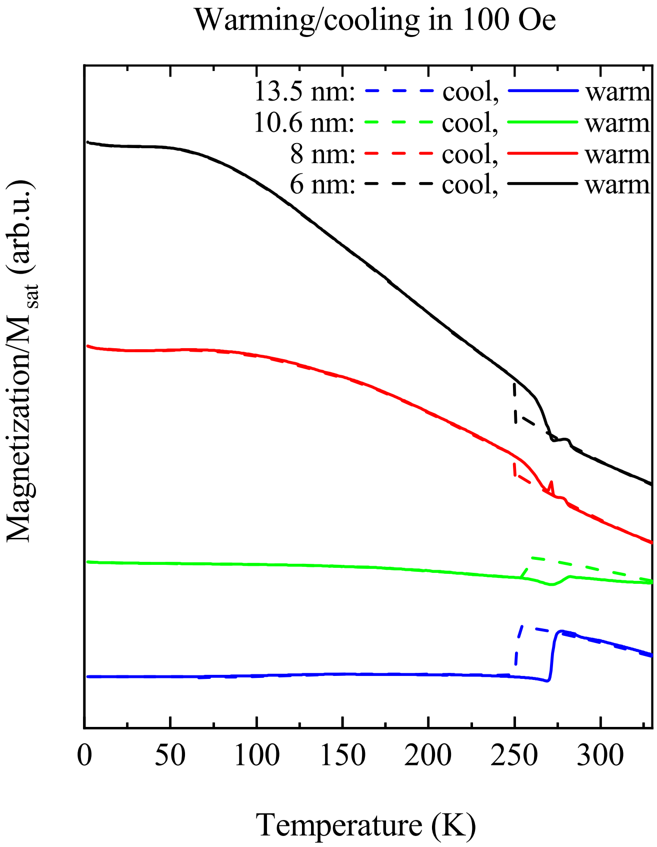

Figure 9: Particle size dependence of the thermal hysteresis effect for the 6, 8, 10.6, and 13.5 nm diameter samples for the 10 mg/mL concentration. The measured magnetization values are normalized by the saturated magnetization value at 330 K and are offset for clarity.

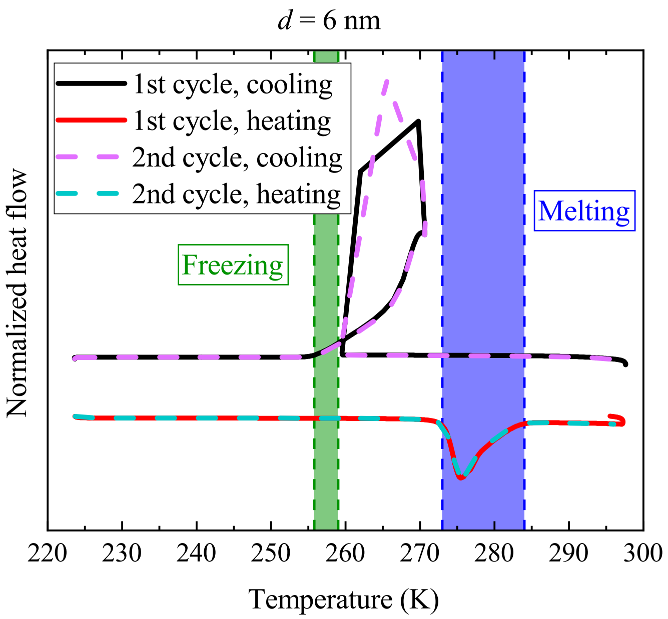

(a)

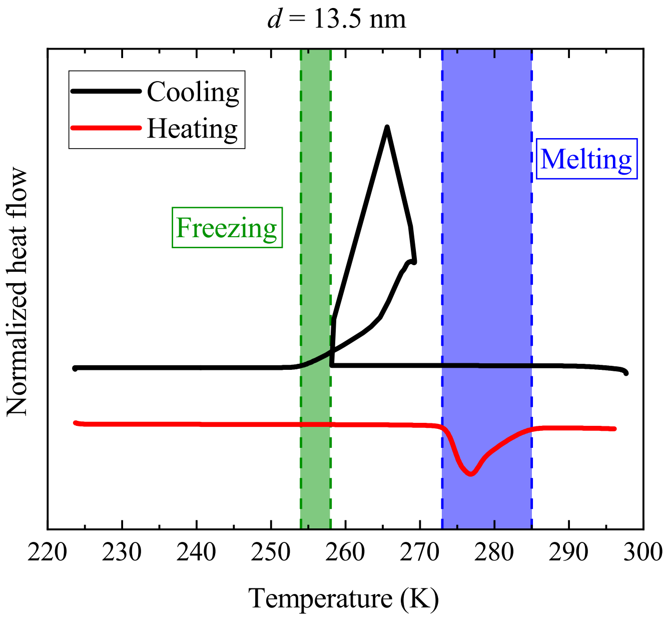

(b)

Figure 10: Normalized heat flow as a function of temperature, measured with the 6-nm (a) and 13.5-nm (b) samples. Curves are arbitrarily shifted for better visibility. Freezing and melting temperature ranges are denoted as shaded areas.
